# Screening of Sepsis Biomarkers Based on Bioinformatics Data Analysis

**DOI:** 10.1155/2022/6788569

**Published:** 2022-09-26

**Authors:** Guibin Liang, Jiuang Li, Shiqian Pu, Zhihui He

**Affiliations:** Department of Critical Care Medicine, The Third Xiangya Hospital, Central South University, Changsha, Hunan, China

## Abstract

**Methods:**

Gene expression profiles of GSE13904, GSE26378, GSE26440, GSE65682, and GSE69528 were obtained from the National Center for Biotechnology Information (NCBI). The differentially expressed genes (DEGs) were searched using limma software package. Gene Ontology (GO) functional analysis, Kyoto Encyclopedia of Genes and Genomes (KEGG) pathway enrichment analysis, and protein-protein interaction (PPI) network analysis were performed to elucidate molecular mechanisms of DEGs and screen hub genes.

**Results:**

A total of 108 DEGs were identified in the study, of which 67 were upregulated and 41 were downregulated. 15 superlative diagnostic biomarkers (CCL5, CCR7, CD2, CD27, CD274, CD3D, GNLY, GZMA, GZMH, GZMK, IL2RB, IL7R, ITK, KLRB1, and PRF1) for sepsis were identified by bioinformatics analysis.

**Conclusion:**

15 hub genes (CCL5, CCR7, CD2, CD27, CD274, CD3D, GNLY, GZMA, GZMH, GZMK, IL2RB, IL7R, ITK, KLRB1, and PRF1) have been elucidated in this study, and these biomarkers may be helpful in the diagnosis and therapy of patients with sepsis.

## 1. Introduction

According to the latest guidelines (sepsis 3.0), sepsis is a syndrome of life-threatening organ dysfunction caused by the imbalance of the body's response to infection. It is one of the main causes of death in patients with critical care medicine (CCM) [1]. In 2020, a study showed that the incidence of sepsis in the world was 677.5 cases per 100 thousand persons [2]. Due to the characteristics of acute onset, complex clinical manifestations, and high mortality of sepsis, early diagnosis of sepsis is the basis of improving the survival rate of sepsis patients [3]. Therefore, finding biomarker is the first step in the early diagnosis of sepsis. However, the sensitivity and specificity of the main laboratory indexes for clinical diagnosis of sepsis are not satisfactory, including C-reactive protein (CRP), interleukin-6 (IL-6), and procalcitonin (PCT) [4]. Although a variety of means have been widely used in the treatment of sepsis in recent years, including active removal of the source of infection, appropriate antibiotic treatment, hemodynamic support, and respiratory support, the mortality of sepsis remains high [5–7]. Finding more effective treatment method for sepsis is an urgent problem to be solved.

At present, the use of bioinformatics technology to mine microarray gene expression data has been widely used to analyze disease-related differentially expressed genes (DEGs), to find key genes, and screen biomarkers related to disease diagnosis, treatment, and prognosis. It was found that fibronectin 1 (FN1), epidermal growth factor (EGF), and transthyretin (TTR) showed considerable diagnostic efficiency for focal segmental glomerulosclerosis, which was based on bioinformatics analysis [8]. According to bioinformatical analyses between rheumatoid arthritis and osteoarthritis, Li et al. identified 15 DEGs which might be therapeutic targets and biomarkers for rheumatoid arthritis treatment [9]. According to bioinformatical analyses, TRPM4 and TRPV2 were considered as two novel prognostic biomarkers and a promising targeted therapy in uveal melanoma (UVM) [10]. After decades of research, the specific pathogenesis and molecular mechanism of sepsis are still unclear. Therefore, finding potential molecular markers of sepsis is very important for developing effective diagnosis and treatment strategies. Benefiting from the development of bioinformatics technology in recent years, researchers can quickly find hub gene clusters through computer programs. Through the comprehensive analysis of sepsis, screening differential genes, establishing gene networks, finding potential key molecular targets, and obtaining early diagnostic markers of sepsis, it is possible to provide a new understanding of the pathogenesis of sepsis and new ideas of the clinical treatment of sepsis. Potential molecular markers are also therapeutic targets for drugs, but the discovery and development of new drugs are long and expensive processes. Therefore, a more effective and rational approach is needed. In this regard, in-silico techniques have been proven to be credible, fast, inexpensive, and effective in tackling the abovementioned problems [11, 12], so many drugs can be better used in the clinic through the in-silico techniques [13–17].

There has been no significant progress in the diagnosis and treatment of sepsis in recent years, and new techniques, including bioinformatics analysis, may contribute to addressing the existing gap between basic research and clinical. In this study, we got sepsis-related mRNA microarray datasets in Gene Expression Omnibus (GEO) and used bioinformatics analysis to screen DEGs in sepsis, so as to provide new gene biomarkers for the diagnosis and treatment of sepsis.

## 2. Materials and Methods

### 2.1. Processing Microarray Data

After retrieving sepsis-related GSE through Gene Expression Omnibus (GEO), the microarray dataset GSE13904, GSE26378, GSE26440, GSE65682, and GSE69528 were downloaded. The sepsis patients (*n* = 1181) and controls (*n* = 168) were collected from GSE13904, GSE26378, GSE26440, GSE65682, and GSE69528.

### 2.2. Screening of DEGs

The original data were downloaded in MINiML format (https://www.ncbi.nlm.nih.gov/). The extracted data were normalized by log2 transformation. The microarray data were normalized by the normalized quantile function of the preprocessing core package in *R* software (version 4.0.3) [18]. According to the annotation information of standardized data in the platform, the probe was converted into gene symbol. The probes matching multiple genes were removed, and the average gene expression value measured by multiple probes was calculated as the final expression value. The differentially expressed mRNA was studied using limma package in *R* software. “*P* < 0.05 and Log (Fold Change) > 3 or Log (Fold Change) < -3” were defined as the threshold for the differential expression of mRNAs. The volcano plot was constructed using the fold change values and *P*-value. Red dots indicated upregulated genes and blue dots indicated downregulated genes. Heat maps were generated using pheatmap package. The heat map showed data in a two-dimensional form, in which colors represented the values.

### 2.3. KEGG and GO Pathway Enrichment Analyses of DEGs

To better understand the gene of mRNA, ClusterProfiler package (version: 4.0.3) in *R* was employed to enrich the KEGG pathway and analyze the GO function of potential targets [18]. The enriched KEGG pathways were selected to demonstrate the primary biological actions of major potential mRNA. The abscissa indicated gene ratio, and the enriched pathways were presented in the ordinate. *Gene ontology (GO) analysis of potential targets of mRNAs.* The biological process (BP), cellular component (CC), and molecular function (MF) of potential targets were clustered based on ClusterProfiler package in *R* software (version: 4.0.3). Colors represent the significance of differential enrichment, the size of the circles represents the number of genes, the larger the circle, the greater the number of genes. In the enrichment result, *P* < 0.05 or FDR <0.05 is considered to be a meaningful pathway.

### 2.4. PPI Network Construction of DEGs

DEGs were uploaded to the STRING database (https://cn.string-db.org/), the interaction score was set to the highest confidence ≥0.4, and PPI analysis was performed. Cytoscape 3.9.2 software (https://cytoscape.org/) was used to visualize PPI network. The MCODE of Cytoscape was used to screen hub genes in the PPI network [19].

## 3. Result

### 3.1. Screening of DEGs

**Figure 1:**
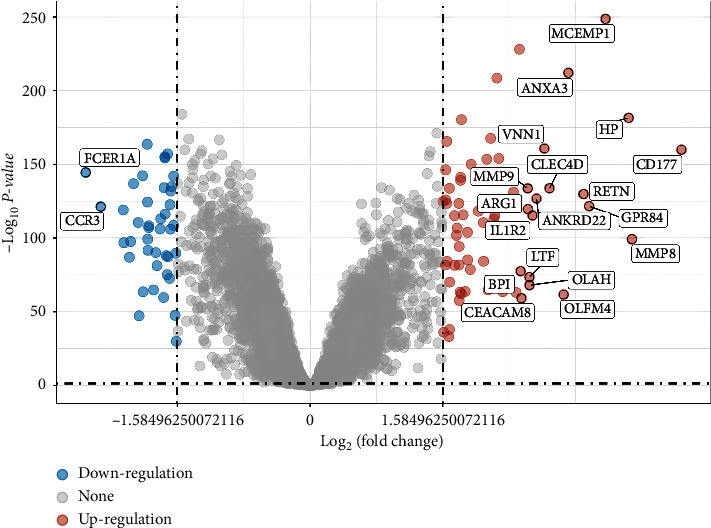
Volcano plot shows the differentially expressed genes of sepsis.

**Figure 2:**
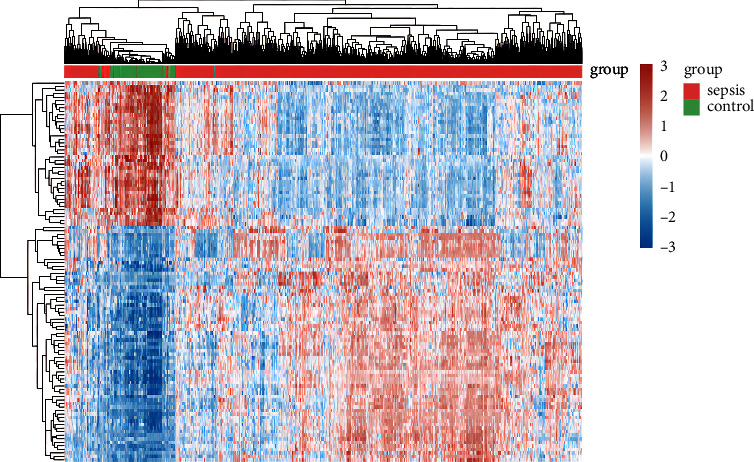
Heat map analysis of identified DEGs between patients with sepsis and uninfected controls. The red color shows the upregulated DEGs, and the blue color shows the downregulated DEGs.

The sepsis patients (*n* = 1181) and controls (*n* = 168) were collected from the gene expression profile GSE13904, GSE26378, GSE26440, GSE65682, and GSE69528. The result of the data preprocessing was assessed by boxplot. A boxplot was made after data normalization. Different colors represented different datasets. Rows represented samples, and columns represented the gene expression values in the samples (Figure S1A). The PCA was the result before batch removal from multiple datasets. Different colors represented different datasets (Figure S1B). The PCA results after batch removal were the intersection of five datasets, which could be used for subsequent analysis (Figure S1C). The volcano plot was utilized to analyze the cluster analysis of the identified DEGs in the GSE13904, GSE26378, GSE26440, GSE65682, and GSE69528 database. Based on the standard of *P* < 0.05 and Log (Fold Change) > 3 or Log (Fold Change) < -3, we screened 108 DEGs, including 67 upregulated genes and 41 downregulated genes (Figure 1). In the GSE13904, GSE26378, GSE26440, GSE65682, and GSE69528 database, the heat map was utilized to analyze the cluster analysis of the identified DEGs, and the results showed a significant difference between the sepsis and the control group (Figure 2). The statistical differences of identified DEGs were shown in Figure S2.

### 3.2. KEGG and GO Pathway Enrichment Analyses of DEGs

**Figure 3:**
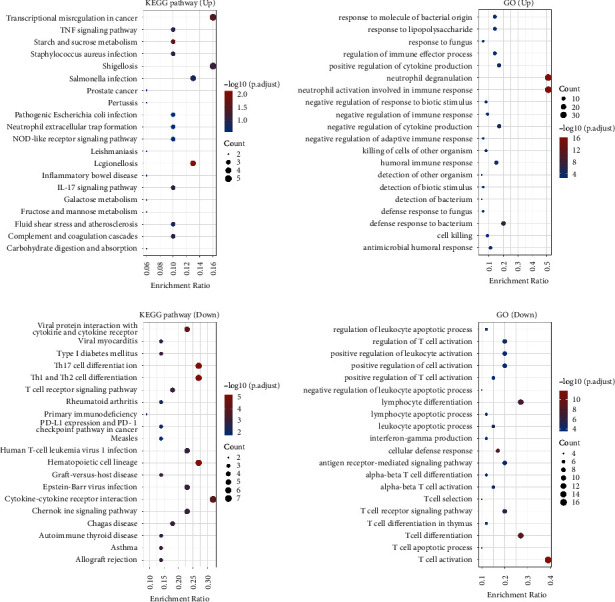
Kyoto Encyclopedia of Genes and Genomes (KEGG) pathway enrichment analysis, Gene Ontology (GO) functional analysis of the up-regulated and down-regulated DEGs, respectively.

Through ClusterProfiler package (version: 4.0.3) in *R* analysis, the KEGG pathways of upregulated DEGs were majorly enriched in transcriptional misregulation in cancer and shigellosis. The GO pathways of upregulated DEGs were majorly enriched in neutrophil degranulation and neutrophil activation involved in immune response. The KEGG pathways of downregulated DEGs were majorly enriched in cytokine-cytokine receptor interaction. The GO pathways of downregulated DEGs were majorly enriched in T cell activation (Figure 3).

### 3.3. PPI Network Construction of DEGs

**Figure 4:**
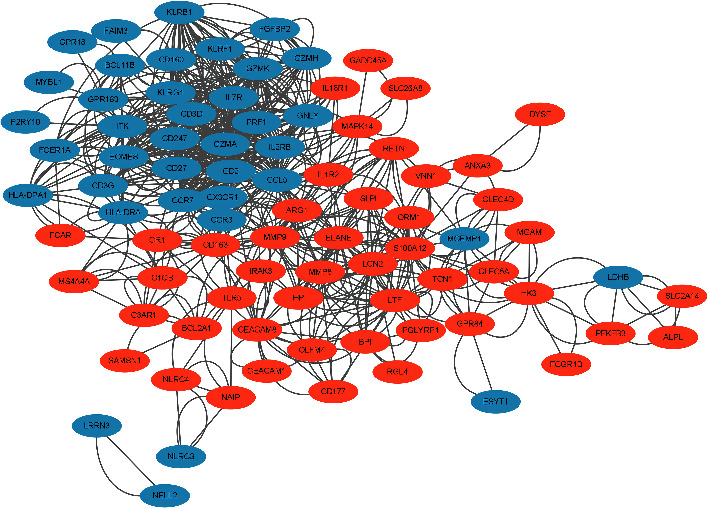
A co-expression module enriched of DEGs. Vertexes correspond to genes and edges correspond to expression correlation. Only the edges with the absolute value of PCC≥0.4 are shown. Up-regulated DEGs are colored in red while down-regulated DEGs are colored in blue.

**Figure 5:**
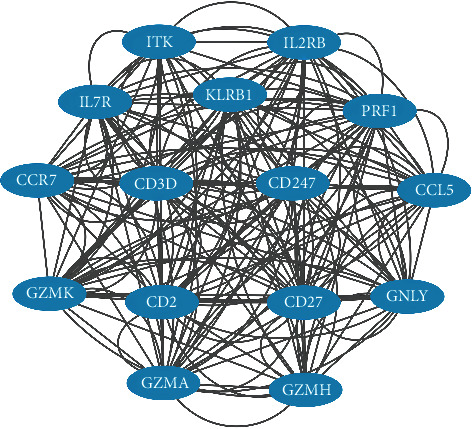
The most significant module obtained from protein-protein interaction (PPI) network.

The screened DEGs were used to construct the PPI network through the STRING database, a co-expression module enriched of DEGs. Vertexes correspond to genes and edges correspond to expression correlation. Only the edges with the absolute value of PCC≥0.4 were shown. Upregulated DEGs were colored in red while downregulated DEGs were colored in blue (Figure 4). Through Cytoscape, we could summarize that CCL5, CCR7, CD2, CD27, CD274, CD3D, GNLY, GZMA, GZMH, GZMK, IL2RB, IL7R, ITK, KLRB1, and PRF1 were hub genes of sepsis (Figure 5). Summary of the hub genes is showed in Table 1.

## 4. Discussion

The pathogenesis of sepsis is very complex, including pathogen invasion, host immune response, and various tissue damage caused by their complex interaction. Sepsis is one of the most common causes of death in intensive care medicine [20]. Although there has been great progress in pathophysiology of sepsis, we still lack the early diagnostic indicator to minimize the incidence rate of sepsis. Bioinformatics analysis enables us to understand the molecular mechanism of disease occurrence and development and provides a new and effective method for the prevention and treatment of sepsis to identify potential diagnostic biomarkers and therapeutic targets.

After the analysis of the sepsis gene expression sequences of GSE13904, GSE26378, GSE26440, GSE65682, and GSE69528 in GEO, we obtained 108 DEGs between the sepsis and control groups, including 67 upregulated genes and 41 downregulated genes. According to KEGG and GO enrichment analyses, upregulated DEGs were enriched in transcriptional misregulation in cancer, shigellosis, neutrophil degranulation, and neutrophil activation involved in immune response, and downregulated DEGs were majorly enriched in cytokine-cytokine receptor interaction and T cell activation. In sepsis, the inflammatory cytokines include tumor necrosis factor-*α* (TNF-*α*), interleukin-18 (IL-18), and interleukin-1*β* (IL-1*β*) [21]. Cytokines can promote the occurrence and development of sepsis through the interaction with cytokine receptors. Using neutralizing antibodies against TNF-*α* can protect mice from mortality during sepsis [22]. Monitoring the level of IL-18 can effectively evaluate the severity and rehabilitation of patients with sepsis [23]. Our study also demonstrated that cytokine-cytokine receptor interaction might regulate the development of sepsis. Martín-Fernández confirmed that neutrophil degranulation is a central event in sepsis physiopathology [24]. Previous study showed that sepsis results in a deluge of proinflammatory and anti-inflammatory cytokines, leading to lymphopenia and chronic immunoparalysis [25]. This was consistent with our study.

There have been many research studies looking for markers for the diagnosis and treatment of sepsis, including microRNAs, long noncoding RNAs, circular RNAs, pancreatic stone protein, and lipopolysaccharide-binding protein [26–30]. In our research, we identified 15 major hub genes (CCL5, CCR7, CD2, CD27, CD274, CD3D, GNLY, GZMA, GZMH, GZMK, IL2RB, IL7R, ITK, KLRB1, and PRF1) in the PPI network, and all of them were downregulated genes in sepsis. CCL5 is a chemokine gene clustered on the chromosome 17, which is a potential target for sepsis [31]. Li *M* identified a gene signature, containing the hub genes CCL5, and established a model that could be used to diagnose patients with sepsis [32]. The protein encoded by CCR7 is a member of the *G* protein coupled receptor family. It can activate B lymphocytes and T lymphocytes, control the migration of memory T cells to inflammatory tissues, and stimulate the maturation of dendritic cells [33]. Yang found that CCR7 was significantly changed in patients with sepsis compared with matched controls [34]. Li Y found that CCR7 might participate in the mechanism of community-acquired pneumonia (CAP) with sepsis [35]. The protein encoded by CD2 is a surface antigen found on all peripheral blood T cells. Another study also found that CD2 is separately identified as the downregulated crucial gene set in sepsis [36]. The protein encoded by CD27 is a member of the tumor necrosis factor receptor superfamily and is also necessary for the production and long-term maintenance of T cell immunity. CD27 can help differentiate the preterm septic neonates from those with risk factors [37]. CD274, also known as PDL1, is a ligand that binds to the receptor PD1. It is usually found on T cells and plays a role in preventing T cell activation [38]. In sepsis mice model, upregulation of PDL1 can delay human neutrophil apoptosis and promote lung injury [39]. CD3D is involved in T cell development and signal transduction, which is an effective tool to identify patients with high or low risk of sepsis after abdominal surgery [40]. Almansa *R* proposed that genes involved in T cell (CD3D) and NK cell immunity were inversely associated with SOFA and mortality [41]. This was consistent with our study. GNLY is a cytolytic antimicrobial peptide (AMP) released from the granules of both cytotoxic T lymphocytes (CTLs) and natural killer (NK) cells, which mainly involved in the occurrence of psoriasis [42]. Zhang *Q* found that the hub genes GNLY may be associated with the prognosis of sepsis [43]. GZMA is a serine protease specific for T cells and NK cells. It may be a necessary common component for cytotoxic T lymphocytes and NK cells to cleave target cells. Garzón-Tituaña verified that GZMA was a key regulator of the inflammatory response during abdominal sepsis [44]. Inhibition of GZMA can reduce inflammation and improve survival during *E*. *coli* sepsis [45]. GZMH is constitutively expressed at high levels in NK cells and plays a pivotal role in NK cell mediated cytolysis [46]. Mediated by perforin and streptolysin O, GZMH can effectively kill host cells. Dead cells show the typical characteristics of programmed cell death, such as reactive oxygen species (ROS) production, mitochondrial depolarization, DNA degradation, and chromatin condensation, while programmed cell death often occurs in sepsis [47]. GZMK is a member of the serine-proteases family, which is mainly expressed by T lymphocytes [48]. In human infectious diseases, GZMK can activate the protease activated receptor-1 (PAR-1) in endothelial cells and induce the production of inflammatory cytokines (TNF-*α*, IL-1, and IL-6) [49]. Turner et al. found that GZMK could affect wound healing by increasing inflammation and hindering epithelialization [50]. IL2RB is part of a receptor signaling complex, and its function is highly pleiotropic. Activation of IL2RB by endogenous IL2 or biased therapeutic stimulation can lead to the expansion of immune cells, especially CD4 +, CD8 + , and NK cells [51]. Targeting IL2RB can reduce acute lung injury caused by sepsis [52]. IL7R is important for the body's immune responses and plays a role in regulating development, differentiation, and survival of T cells [53]. Studies had shown that the level of IL7R in sepsis was significantly reduced, which was consistent with our results [54]. ITK is a member of the Tec family tyrosine kinases and mediates T cell receptor (TCR) signaling pathway [55]. Nadeem A proposed that ITK signaling plays a significant role in sepsis-induced acute lung injury [56]. ITK inhibition may be an effective strategy to terminate sepsis-related acute renal injury [57]. KLRB1, also known as killer cell lectin like receptor B1, is a gene encoding CD161. CD161 is expressed on immune cells (natural killer cells (NK), CD4 +, CD8 + , and other T cell subgroups) [58]. CD161 is also a prognostic biomarker and immunotherapeutic target for low-grade gliomas [59]. Lu *J* identified that KLRB1 was identified as the downregulated crucial gene set in sepsis [36]. PRF1 belongs to the membrane-attack-complex/PRF (MACPF) protein family, which is mainly involved in the particle-dependent killing activity of cytotoxic T lymphocytes (CTL) and NK cells. As a clear marker of the killing ability of immune cells, PRF1 is involved in the establishment of immune homeostasis, pathogen clearance, and tumor monitoring [60].

Our study also demonstrated that cytokine-cytokine receptor interaction might regulate the development of sepsis. Plasma concentrations of specific cytokines TNF-*α*, IL-1*β*, IL-6, and IL-8 are often elevated in patients with sepsis, and cytokine concentrations are associated with the severity and prognosis of sepsis [61]. TNF-*α* can bind to its receptors TNFRSF1A/TNFR1 and TNFRSF1B/TNFBR, which involved in the regulation of a wide spectrum of biological processes including cell proliferation, differentiation, apoptosis, lipid metabolism, and coagulation [62]. IL-6 binds to its membrane-anchored receptor (IL-6R), and the complex of IL-6 and IL-6R recruits a dimer of the transmembrane signal transducer glycoprotein 130 (gp130). Barkhausen et al. found that selective inhibition of IL-6 trans-signaling by sgp130Fc had considerable potential for the treatment of sepsis and related disorders [63]. The activation of macrophage plays a very important role in the immune pathogenesis of sepsis [64]. Macrophages mainly include classically activated M1 macrophages and alternatively activated M2 macrophages [65]. M1 macrophage is associated with hyper-inflammatory phenotype, which mainly secretes IL-1, IL-12, and IL-23 cytokines, and the corresponding cytokine receptors are IL-1R, IL12RB1/2, and IL-23R [65]. M2 macrophage is associated with hypoinflammatory phenotype, which mainly secretes TGF-*β*, VEGF, and EGF cytokines, and the corresponding cytokine receptors are TGFBR3, VEGFR, and EGFR [65]. Among the 15 hub genes mentioned above, CCL5 is a cytokine and CCR7, CD27, CD274, IL2RB and IL7R are cytokine receptors. CCL5 is a chemokine for blood monocytes, memory *T* helper cells, and eosinophils. It functions as one of the natural ligands for the chemokine receptor chemokine (C-C motif) receptor 5 (CCR5) [66]. CCR7 binds to the CCL19 and plays an important role in the trafficking of immune cells as well as cancer metastasis [67]. CD27 binds to ligand CD70 and plays a key role in regulating B cell activation and immunoglobulin synthesis [68]. CD274 is a ligand and binds with the receptor PD1, commonly found on T cells, and acts to block T cell activation [38]. IL2RB binds to ligand IL2, which is involved in T cell-mediated immune responses [69]. IL7R binds to ligand IL7, and the IL7R signals through the JAK/STAT pathway. Loss-of-function mutations and some polymorphisms of the IL7R*α* were associated to immunodeficiency and inflammatory diseases, respectively [70].

This study has some limitations. First, the universality of hub gene in sepsis patients is not verified, which limits the application of the hub gene in sepsis. Second, the involvement of hub gene in the pathogenesis of sepsis needs to be further explored. Finally, we will collect clinical samples to further explore the relationship between the prognosis of patients with sepsis and the expression of CCL5, CCR7, CD2, CD27, CD274, CD3D, GNLY, GZMA, GZMH, GZMK, IL2RB, IL7R, ITK, KLRB1, and PRF1 through relevant experiments in the future.

## 5. Conclusion

In conclusion, a total of 15 hub genes (CCL5, CCR7, CD2, CD27, CD274, CD3D, GNLY, GZMA, GZMH, GZMK, IL2RB, IL7R, ITK, KLRB1, and PRF1) have been screened out as sepsis biomarkers in this study, all of them were downregulated genes in sepsis. Some are cytokines, some are cytokine receptors, and some are proteases or kinases. Cytokine-cytokine receptor interaction might regulate the progress of sepsis. Proteases or kinases are usually expressed in T cell and/or NK cell, participating in sepsis. The hub genes reported in our study may help to unravel some unexplored regulatory pathways, leading to the identification of critical molecular targets for increased diagnosis, prognosis, and drug efficacy in sepsis. Next, we will further elaborate the specific signaling mechanism of these 15 hub genes in sepsis through experiments and find the most suitable genes in clinical practice.

## Figures and Tables

**Table 1:** The summary of hub genes.

Gene symbol	Full name	Function
CCL5	C-C motif chemokine ligand 5	This gene is one of several chemokine genes clustered on the q-arm of chromosome 17. Chemokines form a superfamily of secreted proteins involved in immunoregulatory and inflammatory processes.
CCR7	C-C motif chemokine receptor 7	Receptor for the MIP-3-beta chemokine. Probable mediator of EBV effects on B-lymphocytes or of normal lymphocyte functions.
CD2	CD2 molecule	The protein encoded by this gene is a surface antigen found on all peripheral blood T-cells. The encoded protein interacts with LFA3 (CD58) on antigen presenting cells to optimize immune recognition.
CD27	CD27 molecule	The protein encoded by this gene is a member of the TNF-receptor superfamily.
CD274	CD274 molecule	This gene encodes an immune inhibitory receptor ligand that is expressed by hematopoietic and non-hematopoietic cells, such as T cells and B cells and various types of tumor cells.
CD3D	CD3d molecule	The protein encoded by this gene is part of the T-cell receptor/CD3 complex (TCR/CD3 complex) and is involved in T-cell development and signal transduction.
GNLY	Granulysin	The product of this gene is a member of the saposin-like protein (SAPLIP) family and is located in the cytotoxic granules of T cells, which are released upon antigen stimulation.
GZMA	Granzyme A	GZMA is a T cell- and natural killer cell-specific serine protease that may function as a common component necessary for lysis of target cells by cytotoxic T lymphocytes and natural killer cells.
GZMH	Granzyme H	This protein is reported to be constitutively expressed in the NK (natural killer) cells of the immune system and may play a role in the cytotoxic arm of the innate immune response by inducing target cell death and by directly cleaving substrates in pathogen-infected cells.
GZMK	Granzyme K	This gene product is a member of a group of related serine proteases from the cytoplasmic granules of cytotoxic lymphocytes.
IL2RB	Interleukin 2 receptor subunit beta	Receptor for interleukin-2. This beta subunit is involved in receptor mediated endocytosis and transduces the mitogenic signals of IL2. Probably in association with IL15RA, involved in the stimulation of neutrophil phagocytosis by IL15.
IL7R	Interleukin 7 receptor	The protein encoded by this gene is a receptor for interleukin 7 (IL7).
ITK	IL2 inducible *T* cell kinase	This gene encodes an intracellular tyrosine kinase expressed in T-cells. The protein contains both SH2 and SH3 domains which are often found in intracellular kinases. It is thought to play a role in T-cell proliferation and differentiation.
KLRB1	Killer cell lectin like receptor B1	Plays an inhibitory role on natural killer (NK) cells cytotoxicity.
PRF1	Perforin 1	This gene encodes a protein with structural similarities to complement component C9 that is important in immunity.

## Data Availability

The dataset(s) supporting the conclusions of this article is available in the NCBI GEO database, GSE13904, GSE26378, GSE26440, GSE65682, and GSE69528 https://www.ncbi.nlm.nih.gov/.

## Supplementary Materials

Supplementary Materials Table S1 Baseline characteristics of patients. Table S2 108 differentially expressed mRNAs after reducing data dimensions. Figure S1 Batch effect processing between different datasets. Different colors represent different datasets. Rows represent samples, and columns represent the gene expression values in the samples (A). PCA results before batch removal for multiple datasets. Different colors represent different datasets. (B). The PCA results after batch removal were the intersection of five datasets, which can be used for subsequent analysis (C). Figure S2 The expression distribution of CCL5 gene in different patients. The abscissa represents different groups of samples, and the ordinate represents the expression distribution of the gene, different colors represent different groups, and top-left represents the significance *p*-value. ∗*p* <  0.05, ∗∗*p* <  0.01,∗∗∗p  <  0.001, and asterisks (∗) stand for significance levels. The statistical difference of two groups was compared through the Wilcox test.
