# Supplementary figures and images for: Screening of Sepsis Biomarkers Based on Bioinformatics Data Analysis
Source: J Healthc Eng. 2022 Sep 26;2022:6788569. doi: 10.1155/2022/6788569 (PMC9529510; doi:10.1155/2022/6788569)

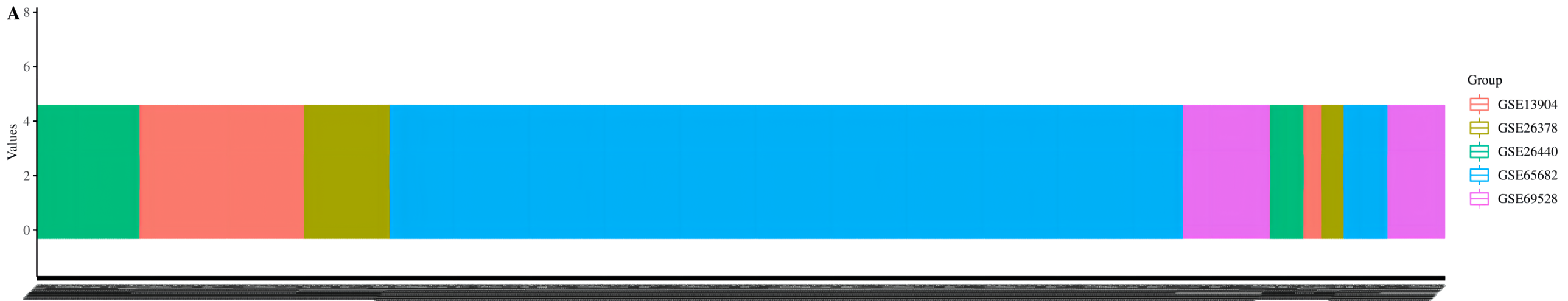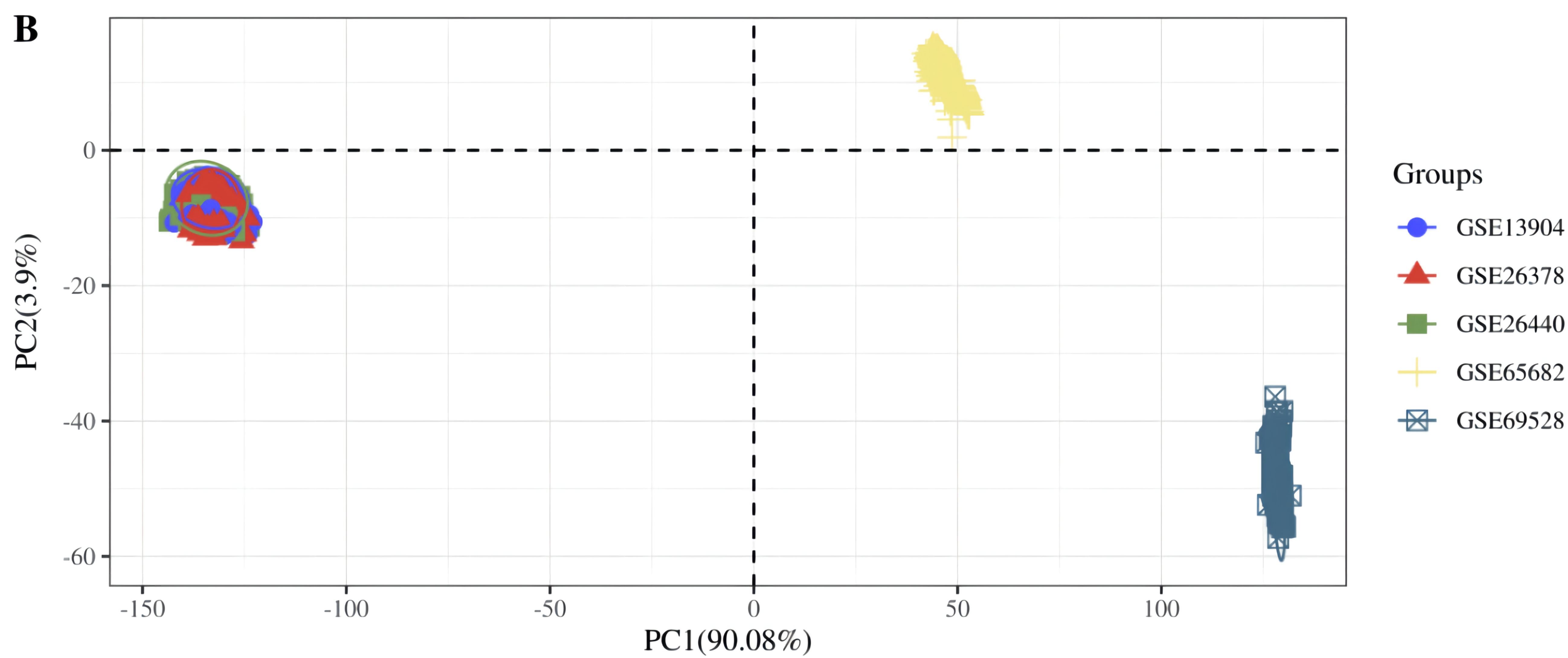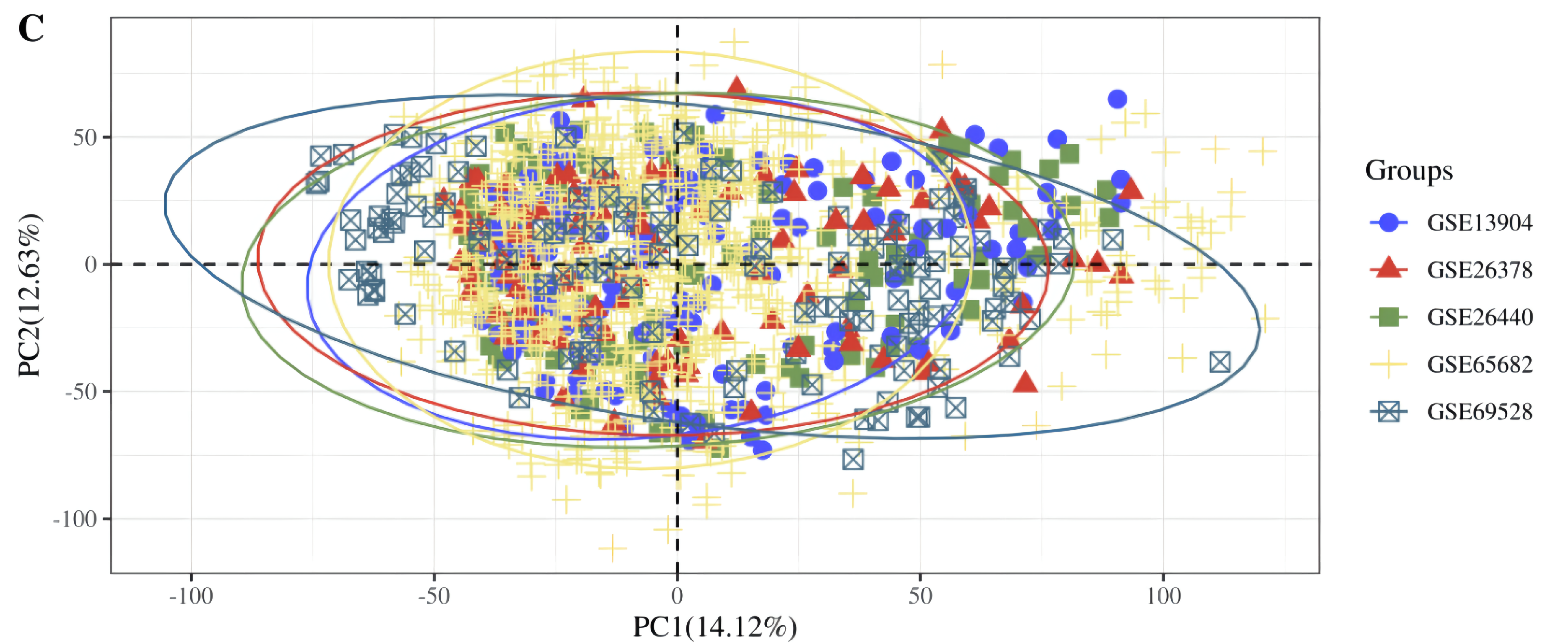

Supplement: Supplementary Materials — Table S1 Baseline characteristics of patients. Table S2 108 differentially expressed mRNAs after reducing data dimensions. Figure S1 Batch effect processing between different datasets. Different colors represent different datasets. Rows represent samples, and columns represent the gene expression values in the samples (A). PCA results before batch removal for multiple datasets. Different colors represent different datasets. (B). The PCA results after batch removal were the intersection of five datasets, which can be used for subsequent analysis (C). Figure S2 The expression distribution of CCL5 gene in different patients. The abscissa represents different groups of samples, and the ordinate represents the expression distribution of the gene, different colors represent different groups, and top-left represents the significance p-value. ∗p < 0.05, ∗∗p < 0.01,∗∗∗p < 0.001, and asterisks (∗) stand for significance levels. The statistical difference of two groups was compared through the Wilcox test. [file 6788569.f1.zip › Figure S1.pdf]

wilcox.tests p=3.3e-65

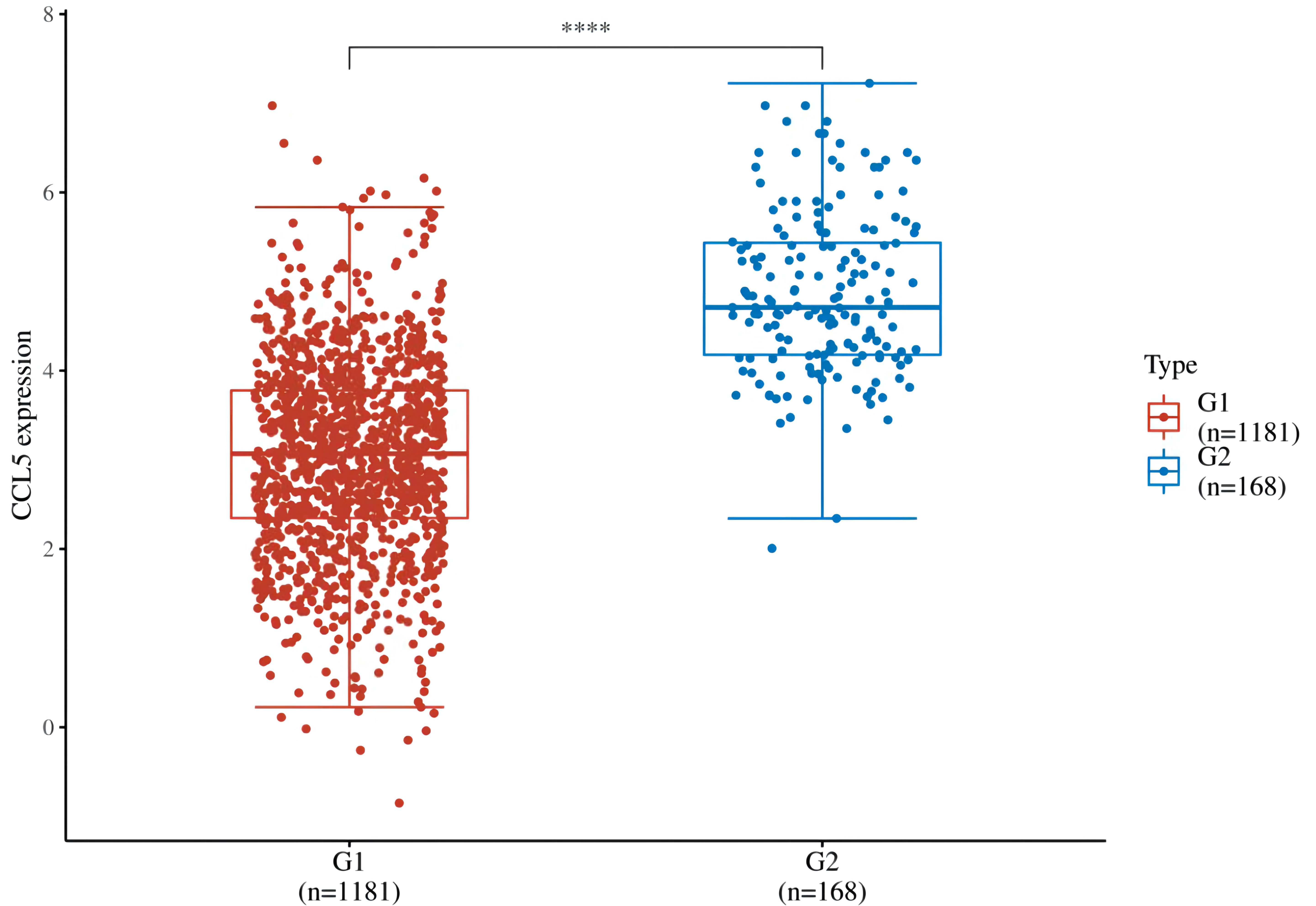

Supplement: Supplementary Materials — Table S1 Baseline characteristics of patients. Table S2 108 differentially expressed mRNAs after reducing data dimensions. Figure S1 Batch effect processing between different datasets. Different colors represent different datasets. Rows represent samples, and columns represent the gene expression values in the samples (A). PCA results before batch removal for multiple datasets. Different colors represent different datasets. (B). The PCA results after batch removal were the intersection of five datasets, which can be used for subsequent analysis (C). Figure S2 The expression distribution of CCL5 gene in different patients. The abscissa represents different groups of samples, and the ordinate represents the expression distribution of the gene, different colors represent different groups, and top-left represents the significance p-value. ∗p < 0.05, ∗∗p < 0.01,∗∗∗p < 0.001, and asterisks (∗) stand for significance levels. The statistical difference of two groups was compared through the Wilcox test. [file 6788569.f1.zip › Figure S2.pdf]
